# The relationship between HMGB1 and autophagy in the pathogenesis of diabetes and its complications

**DOI:** 10.3389/fendo.2023.1141516

**Published:** 2023-03-29

**Authors:** Kun Yang, Feng Cao, Weili Wang, Zhenyu Tian, Lu Yang

**Affiliations:** ^1^ College of Traditional Chinese Medicine, Tianjin University of Traditional Chinese Medicine, Tianjin, China; ^2^ College of Acupuncture and Massage, Beijing University of Chinese Medicine, Beijing, China; ^3^ Department of Acupuncture, Haidian District Shuangyushu Community Health Service Center, Beijing, China; ^4^ Institute of Basic Research in Clinical Traditional Chinese Medicine, China Academy of Chinese Medical Sciences, Beijing, China; ^5^ The Key Laboratory of Cardiovascular Remodeling and Function Research, Chinese Ministry of Education, Chinese National Health Commission and Chinese Academy of Medical Sciences, The State and Shandong Province Joint Key Laboratory of Translational Cardiovascular Medicine, Department of Cardiology, Qilu Hospital, Cheeloo College of Medicine, Shandong University, Jinan, China

**Keywords:** HMGB1, autophagy, diabetes, diabetic complication, TCM

## Abstract

Diabetes mellitus is a chronic metabolic disorder characterized by elevated blood glucose levels and has become the third leading threat to human health after cancer and cardiovascular disease. Recent studies have shown that autophagy is closely associated with diabetes. Under normal physiological conditions, autophagy promotes cellular homeostasis, reduces damage to healthy tissues and has bidirectional effects on regulating diabetes. However, under pathological conditions, unregulated autophagy activation leads to cell death and may contribute to the progression of diabetes. Therefore, restoring normal autophagy may be a key strategy to treat diabetes. High-mobility group box 1 protein (HMGB1) is a chromatin protein that is mainly present in the nucleus and can be actively secreted or passively released from necrotic, apoptotic, and inflammatory cells. HMGB1 can induce autophagy by activating various pathways. Studies have shown that HMGB1 plays an important role in insulin resistance and diabetes. In this review, we will introduce the biological and structural characteristics of HMGB1 and summarize the existing knowledge on the relationship between HMGB1, autophagy, diabetes, and diabetic complications. We will also summarize potential therapeutic strategies that may be useful for the prevention and treatment of diabetes and its complications.

## 1 Introduction

Diabetes mellitus (DM) is a metabolic disease characterized by chronic hyperglycemia resulting from the complex interplay of genetic, environmental, and other factors that impair pancreatic β cell function and peripheral insulin resistance, leading to glucose metabolism disorders and chronic low-grade inflammation (1). Diabetes is the third leading cause of death worldwide after cardiovascular disease and cancer. The International Diabetes Federation (IDF) reports that there are 537 million cases of diabetes globally and an annual mortality rate of 6.7 million due to diabetes or its complications. In China, the number of DM patients exceeds 116.4 million, ranking highest in the world. The increasing number of patients imposes significant burdens on the healthcare system and the economy, affecting individuals, families, and society at large (2). DM is classified into four main categories: type I diabetes, type II diabetes, gestational diabetes, and other specific types. Persistent hyperglycemia and longterm metabolic dysfunction give rise to a range of complications, and studies have demonstrated a strong association between hyperglycemia and microangiopathy across all types of diabetes. Diabetes induces nerve damage to the heart and leads to cardiac hypertrophy, resulting in diabetic cardiomyopathy (DCM) (3, 4); impairs the formation of retinal blood vessels, causing visual impairment or blindness, which leads to diabetic retinopathy (DR) (5, 6); results in scarring and fibrosis in kidney tissue, causing diabetic nephropathy (DN) (7, 8); affects the nervous system, causing diabetic neuropathy (9); and often leads to numbness, tingling, and pain, resulting in diabetic foot disease. These chronic complications are the leading causes of morbidity and mortality in patients with diabetes (10).

In recent years, researchers have studied the underlying mechanisms of diabetes development and progression, as well as its complications, to develop effective therapeutic strategies to address these pathologies. Among several molecular mechanisms, autophagy is closely associated with diabetes and plays an important role in its development. Therefore, elucidating the molecular mechanisms associated with autophagy in diabetes can aid in the development of new therapeutic options. High-mobility group protein B1 (HMGB1) is a conserved nonhistone nuclear protein that is widely distributed in tissues and organs throughout the body. HMGB1 is involved in replication, recombination, transcription, and DNA repair processes and has been shown to play an important role in autophagy regulation. Activation of HMGB1 and autophagy are associated with the development of various diseases, such as diabetes, tumors, and cardiovascular and cerebrovascular diseases (11). In this paper, we review recent advances in the role of HMGB1 and autophagy in diabetes and its complications, providing new ideas for the treatment and research of this disease.

## 2 Autophagy, diabetes and diabetic complications

**Figure 1:**
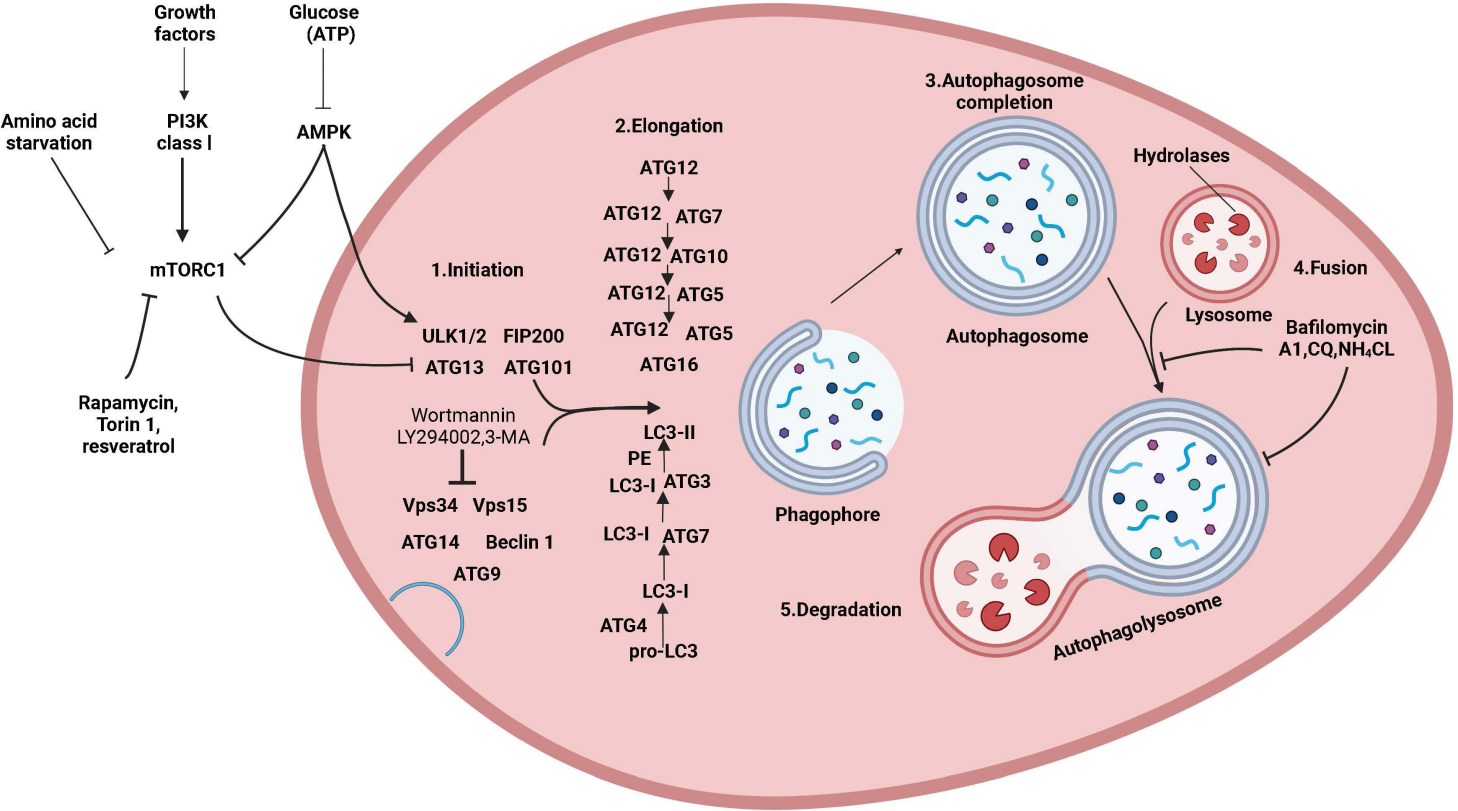
The mechanism of autophagy and its regulation. Molecular circuits and signaling pathways that regulate autophagy. Autophagy is a complex self-degradation process involving the following key steps: the control of Beclin-1/VPS34-mediated formation of phagosomes on the ER and other membranes in response to stress signaling pathways; Atg5-Atg12 conjugation and interaction with Atg16L and the multimerization of the phagosome; LC3 processing and insertion into the extended phagosome membrane; the capture of random or selective targets for degradation to complete the autophagosome while ATG4 recycles some LC3-II/ATG8; and autophagosome fusion with lysosomes and proteolytic degradation of the engulfed molecules by lysosomal proteases. Autophagy is regulated by important signaling pathways in cells, including stress signaling kinases such as JNK-1, which promotes autophagy by phosphorylating Bcl-2, thereby promoting the interaction of Beclin-1 with VPS34. Perhaps the core signaling molecule that determines the level of autophagy in cells is the kinase mTOR, which can mediate its effect on autophagy by inhibiting the ATG1/Ulk-1/-2 complex in the earliest stage of lipid bilayer phagophore formation. mTOR is key to integrating metabolic factors, growth factors, and energy signals associated with autophagy. On the one hand, mTOR inhibits autophagy when nutrients are abundant, and on the other hand, mTOR signaling stimulates growth-promoting activities, including protein translation. Autophagy is induced by hypoxia and low cytoplasmic ATP levels, which feed through REDD1 and AMP kinase to inhibit mTOR activity by reducing RhebGTPase activity. Conversely, increased growth factor signaling through the insulin receptor and its adapter IRS1 and other growth factor receptors that activate class I PI3 kinase and Akt inhibits autophagy and promotes mTOR activity by inhibiting TSC1/TSC2 and increasing RhebGTPase activity.

Autophagy is a conserved and tightly regulated lysosomal pathway for the degradation of aberrant cellular protein aggregates and damaged organelles. It is a self-protective process that digests damaged organelles or misfolded proteins *via* the intracellular lysosomal pathway to maintain intracellular homeostasis. The morphological process of autophagy consists of several successive stages: initiation, nucleation, elongation, closure, maturation, and finally, the degradation and export of materials to the cytoplasm (**Figure 1**). Autophagy is a complex and diverse process, and it can be classified into three main types based on different mechanisms and pathways for the removal of cytoplasmic components: macroautophagy, microautophagy, and molecular chaperone-mediated autophagy (12). Additionally, there are other specific types of autophagy, such as secretory autophagy, starvation-induced degradation of nascent granules, Golgi membrane-associated degradation, and vesicular autophagy, all of which are associated with hormone-secreting cells (13). Macroautophagy is the most widespread and involves the formation of double-membrane vesicles called autophagosomes, which randomly isolate cytoplasmic components before fusing with lysosomes. Autophagy can also target specific organelles or molecules (selective autophagy), and each type of selective autophagy has a name, such as mitochondrial autophagy, endoplasmic reticulum autophagy, peroxisomal autophagy, granular autophagy, ribosomal autophagy, and lipophagy. Selective autophagy has not only the same mechanisms as macroautophagy but also specific mechanisms and functions depending on the target organelle or molecule (14).

### 2.1 Autophagy and diabetes

**Figure 2:**
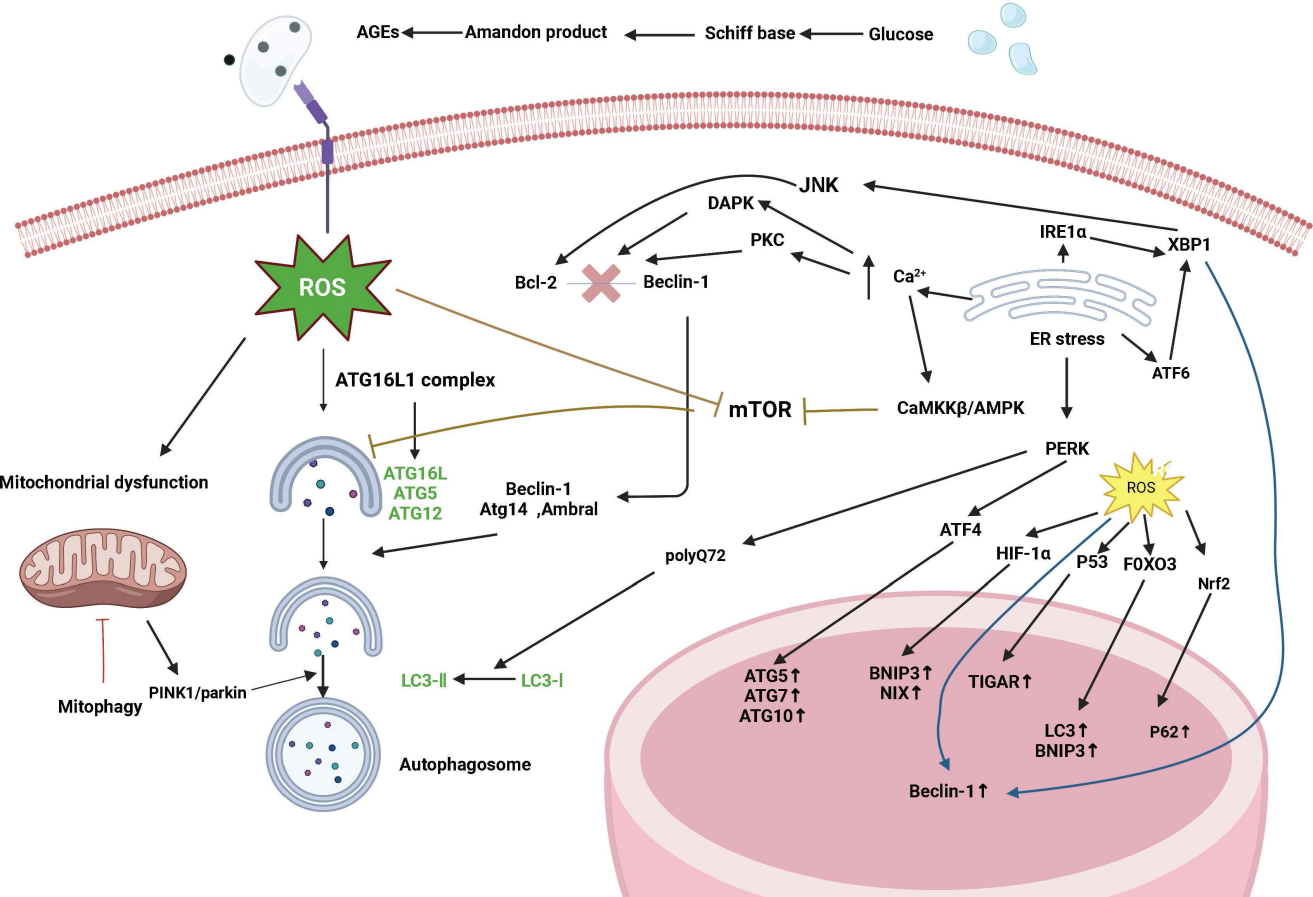
Signaling pathways are regulated by autophagy in diabetes. Diabetes affects autophagy through the following pathways: oxidative stress, endoplasmic reticulum stress, mTOR-dependent signaling pathway, and the AMPK pathway. Autophagy maintains ROS levels by removing partially depolarized mitochondria, and ROS can activate autophagy by inhibiting mTOR, increasing Beclin-1 expression, and converting LC3-I to LC3-II. ROS can act as signaling molecules that activate JNK-1, and large amounts of ROS lead to the opening of the mitochondrial permeability transition pore, which disrupts mitochondrial membrane potential and leads to the onset of PINK1/parkin-mediated autophagy. ROS can also induce autophagy through regulation at the transcriptional level (in the nucleus) and translational level (in the cytoplasm), and an increase in ROS activates the transcription factors HIF-1α, p53, FOXO3, and Nrf2 and promotes the transcription of BNIP3, NIX, TIGAR, LC3, and p62. Endoplasmic reticulum stress induces autophagy through the UPR pathway and endoplasmic reticulum Ca2+ transport. The IRE1α pathway activates the cJun-N-terminal kinase (JNK) pathway and modification of x-box binding protein 1 (XBP1) mRNA by a shift mutation. The ATF6 pathway regulates other UPR members including XBP1. The PERK pathway affects autophagy by inducing the transcription of autophagy-related genes such as ATG5, ATG7, and ATG10 through ATF4. The PERK pathway also facilitates the conversion of LC3-I to LC3-II induced by polyglutamine 72 (PolyQ72). In the early stages of endoplasmic reticulum stress, activated IRE1α phosphorylates Bcl-2, which is involved in the induction of autophagy, *via* the JUK pathway, leading to the separation of Bcl-2 from Beclin-1. In addition, another participant of the XBP-1-IRE1α pathway promotes Beclin-1 transcription. ATF6 is indirectly involved in autophagy by inducing XBP-1 transcription. Under ER stress, excessive Ca2+ entry into the cytoplasm induces autophagy through three different mechanisms: Stimulation of the CamKK/AMPK-dependent pathway leading to mTOR inhibition; Activation of death-associated protein kinase (DAPK) by participating in the phosphorylation of Beclin-1; and activation of the PKC pathway leading to Bcl2-Beclin-1 complex segregation.

The pathogenesis of type 1 and type 2 diabetes is rooted in the imbalance of glucose homeostasis. Although the two forms of diabetes differ in their pathogenesis, islet β-cell failure and death are key factors in diseases. This leads to a decrease in insulin production capacity, resulting in hyperglycemia. Previous studies have shown that islet β-cell apoptosis plays a major role in the development of diabetes in different diabetes models. Increasing levels of islet β-cell autophagy can help maintain and protect the number, structure, and function of islet β cells (as illustrated in **Figure 2**). Therefore, inhibiting excessive apoptosis in pancreatic β cells is crucial for the treatment of DM. Studies have confirmed that impaired autophagy regulation is associated with pancreatic β-cell failure in type 2 diabetes (T2DM) (15). However, the mechanism of autophagy in type 1 diabetes remains unclear. Autophagy helps control the development, function, and survival of β cells by removing misfolded proteins and damaged organelles. A unique mechanism to maintain eukaryotic cell stability is involved in the degradation of pancreatic β-cell secretory granules to maintain normal physiological levels (16) and plays an important role in regulating pancreatic β-cell apoptosis. Some scholars have found experimental evidence of impaired autophagy in islet β cells in type 1 diabetes (17), providing a reference for studying the mechanism of autophagy in type 1 diabetes.

Autophagy has three major roles in pancreatic β cells. First, autophagy maintains the homeostasis of pancreatic β cells and promotes insulin synthesis and secretion. Islet β cells are sensitive to ERS, and autophagy can clear damaged proteins/protein aggregates. Autophagy can maintain the proinsulin homeostasis in islet β cells. Studies have shown that there are a large number of mutant proinsulin and aggregates in the islet β cells of Beta-Atg7-autophagy-deficient mice (18). The high-glucose environment stimulates islet β cells to synthesize more insulin to compensate, consumes large amounts of ATP, and increases the burden on mitochondria; in addition, the high-glucose environment can generate large amounts of ROS to damage the mitochondrial electron transport chain, resulting in mitochondrial damage (19), and clears the affected cells through autophagy. Damaged mitochondria are particularly important for maintaining the homeostasis of islet β cells. Some scholars fed a high-fat diet (HFD) to autophagy-deficient mice and observed a large number of damaged mitochondria in islet β cells. Increasing autophagy can significantly reduce the damage to HFD-fed mice. Mitochondria and insulin synthesis and secretion were increased significantly. Pasquier et al. conducted granule degradation experiments on rats and found that stress-induced degradation of insulin granules interfered with the protective effect of autophagy on islet β cells, leading to the premature death of islet β cells (20). In a low-glucose environment, autophagy can degrade insulin granules and reduce insulin secretion by pancreatic β cells, which is very important for maintaining normal blood sugar during fasting. Second, autophagy can inhibit apoptosis in islet β cells and promote their proliferation; apoptosis is a highly controlled and evolutionarily conserved form of programmed cell death. An increase in autophagy can inhibit stress-induced cell damage, prevent further cell deterioration, prevent cell apoptosis and promote cell proliferation (21, 22). A study using asprosin to stimulate MIN6 cells showed that inhibiting autophagy could induce apoptosis in islet β cells (23). Palmitic acid (PA) reduces the expression of mitogen-activated protein kinase phosphatase 5 (MKP-5) and inhibits autophagy in mouse islet β cells, and overexpressing MKP-5 inhibits PA-induced apoptosis by promoting autophagic death, dysfunction, inflammation and oxidative stress (24). In addition, IL-6 can reduce ROS and improve the viability of islet β cells by promoting autophagy to resist apoptosis and stimulating mitophagy in these cells (25). Therefore, autophagy plays an active role in protecting pancreatic β cells from apoptosis and promoting proliferation. In addition, autophagy can reduce the aggregation of islet amyloid polypeptide (IAPP) in islet β cells; IAPP is a peptide substance composed of 37 amino acid residues that is mainly present in islet β cells (26). Under various physiological (such as pregnancy and aging) or pathological (such as inflammation, DM, and pathological stress) conditions, the expression of IAPP in islet β cells increases, clearance decreases, and a large amount of IAPP aggregates to form islet amyloids, resulting in islet β-cell dysfunction and death (27). Autophagy can degrade IAPP aggregates in islet β cells, thereby reducing islet β cell damage and providing amino acids as raw materials for the synthesis of other peptides (28).

### 2.2 Autophagy and insulin resistance

Insulin resistance refers to a state in which the body’s insulin cannot effectively exert its normal physiological effects. This state is characterized by decreased sensitivity and responsiveness of insulin-acting target tissues, such as the liver, skeletal muscle, and fat, to insulin. As a result, glucose and lipid metabolism disorders may occur in the body. The pathogenesis of insulin resistance is complex and involves multiple molecular factors, such as insulin, insulin receptors, and signal transduction pathways. Additionally, genetics play a crucial role in the development of insulin resistance (29).

Research has shown that insulin resistance is a critical link in the development of T2DM, as it reflects the body’s sensitivity to insulin and is a significant factor that influences the onset and progression of T2DM (30). Although the pathogenesis of type 1 DM (T1DM) and T2DM is entirely different, studies have indicated an increasing prevalence of insulin resistance in T1DM patients (31). Moreover, researchers (32) have shown that insulin resistance in T1DM rats is associated with triglyceride accumulation in skeletal muscle, as shown by insulin tolerance tests. Additionally, there is a lack of research exploring the relationship between insulin resistance and diabetic complications, and further investigation into the underlying mechanisms linking the two is warranted.

A connection between insulin resistance and autophagy has been established. Yang proposed that defects in hepatic autophagy in obesity can lead to insulin resistance (33). The researchers found that regulatory autophagy-related (ATG) proteins were expressed at lower levels in insulin-resistant mice, and liver- or adipose-specific ATG-knockout mice showed abnormal insulin secretion and systemic insulin resistance. Further supporting the role of autophagy in islet signaling, studies have shown that beclin1 mutations induce overactive autophagy and increased hepatic insulin signaling in mice (13). Scott demonstrated that a deficiency in the autophagy gene ATG16L1 in mouse embryonic fibroblasts (MEFs) caused the loss and degradation of the sensitive insulin receptor protein (Scott 34). This effect was mediated by the novel E3Ub ligase complex KLHL9/KLHL13/CUL3, which induced IRS1-targeted degradation, leading to insulin resistance. Because insulin resistance is associated with IRS1 dysfunction, the mechanism by which autophagy deficiency triggers IRS1 degradation highlights the potential for targeting autophagy and/or KLHL9/KLHL13/CUL3-mediated targeted degradation of factors associated with insulin resistance to restore the effects of insulin.

Insulin is well known to inhibit autophagy by activating mTORC1, which leads to the phosphorylation and repression of ULK1 and inactivation of FOXO transcription factors, thus integrating insulin signaling with gene expression. Insulin resistance occurs when insulin fails to sufficiently stimulate glucose uptake in muscle and inhibit hepatic glucose production. This condition arises due to reduced effects of insulin on skeletal muscle, the liver, and adipose tissue, and reduced phosphorylation of AKT on serine and threonine residues regulates insulin activity. AKT activates mTORC1 and inhibits FOXO1/3, thereby inhibiting autophagy. Defects in insulin signaling lead to reduced activation of mTORC1 and hyperactivation of FOXO proteins. Numerous studies have shown that defects in autophagy are an underlying factor in the development of insulin resistance in insulin-target tissues. Therefore, the attenuation of autophagy may induce negative feedback inhibition of insulin signaling, which can overcome the inhibitory effect of insulin on autophagy (35). Even in insulin resistance, autophagy can protect pancreatic β cells.

### 2.3 Autophagy and diabetic complications

During the process of longterm abnormal blood sugar elevation in diabetic patients, various tissues and cells in the body develop an oxidative stress response due to glucose metabolism disorder, which damages the endothelial tissue and activates inflammatory responses and abnormal blood coagulation mechanisms. This can eventually lead to diabetic complications. As the disease progresses, the degree of damage to multiple organs throughout the body worsens, affecting the cardiovascular system, retina, nervous system, kidney, lung, and cognitive function. Compared to the metabolic abnormalities caused by diabetes itself, diabetes-related complications pose a greater threat to patient health.

Cardiovascular complications are one of the main causes of death in diabetic patients. Changes in glucose and lipid metabolism in cardiomyocytes, endoplasmic reticulum stress, oxidative stress, imbalances in Ca2+ homeostasis and other cellular homeostasis, insulin signaling, renin-vascular tension, activation of the hormone system, and autophagy are all involved in the occurrence and development of DCM. The regulation of autophagy in response to cardiac energy stress is mediated by a network system consisting of adenosine-5-phosphate-activated protein kinase (AMPK) and insulin signaling pathways. Moreover, studies have shown that Beclin1 is an important gene for regulating cardiac autophagy (36). Zhang et al. found that liraglutide, a hypoglycemic drug, could prevent diabetes-induced myocardial damage by activating the Sirt1/AMPK signaling pathway (37).

DR is a microvascular complication of diabetes. Recent studies have shown that autophagy plays an important role in the formation of new blood vessels and vascular leakage in DR. Autophagy can regulate the secretion of vascular endothelial growth factor (VEGF) and alleviate corresponding lesions. Mao et al. suggested that Sirtuin 3 (Sir3) could inhibit expression of the migration-related factors MMP-2 and MMP-9 and the angiogenic factors VEGF, HIF-1a, and IGF-1 in HRECs. A study showed that Sir3 overexpression could promote the expression of LC3I and LC3II to promote autophagy, and the expression of VEGF and HIF-1α can be reduced in the late stage of autophagy. Therefore, Sir3 may inhibit angiogenesis in HRECs through autophagy (38). Another study by 39 showed that Müller cells stimulated by HOG-LDL had activated AMPK and increases in the levels of the autophagy-related proteins ATG-5, Beclin-1, and LC3II/LC3I and the expression of the apoptosis-related protein caspase-3. This finding suggests that autophagy activation can lead to apoptosis.

DN is one of the most serious complications of diabetes. Glomerular endothelial cell damage and podocyte autophagy levels around glomerular vessels play important roles in the development of DN. One study showed that salvianolic acid A weakened ROS production induced by AGEs through the AGE-receptor for advanced glycosylation end products (RAGE)-Nox4 axis, thereby promoting the expression of Atg5, Atg7, Atg12, and LC3II/LC3I and inhibiting the expression of p62 to ameliorate high-glucose (HG)-induced autophagy disorders in endothelial cells and inhibiting the progression of DN (40). Other studies have demonstrated that autophagy activation in glomerular endothelial cells protects renal function. Several renoprotective drugs have been shown to promote the expression of autophagy-related proteins such as LC3II and Beclin-1 through the mTOR/AMPK pathway, enhancing autophagy and protecting podocytes (41, 42).

## 3 HMGB1, diabetes and diabetic complication

### 3.1 HMGB1 and diabetes

HMGB1 is implicated in the pathogenesis of diabetes, and elevated levels of HMGB1 have been observed in diabetic patients and animal models (43–45). HMGB1 is passively released by injured pancreatic β cells or actively secreted by DCs and macrophages that infiltrate the islets. In type 1 diabetes, extracellular HMGB1 enhances autoimmune progression by destabilizing regulatory T cells (46). Patients with T2DM have a subclinical systemic inflammatory state, and HMGB1, a late mediator of inflammation, is an important mediator of the pathogenesis of T2DM. Early inflammation in adipose tissue and islets leads to necrosis in adipose-derived stromal cells and islet cells. Necrotic cells release HMGB1, which activates TLRs and RAGE on macrophages and dendritic cells. The activation of TLRs and RAGE leads to the translocation of NF-κB into the nucleus and promotes the expression of inflammatory genes, contributing to the secretion of proinflammatory cytokines, including HMGB1. In addition, activated macrophages and dendritic cells actively secrete HMGB1, which in turn exacerbates necrosis in adipose tissue and pancreatic islets (47).

HMGB1 is involved in the onset of diabetic complications, including diabetic vascular disease such as diabetic heart disease, and diabetic heart disease is considered by some scholars to be a mitochondrial disease (48). While HMGB1 is a key regulator of mitochondrial autophagy, redox-induced HMGB1 translocation can result in HMGB1 binding to late glycosylation end products, leading to sustained proinflammatory pathway activation, enhanced myocardial injury, and the regulation of heat shock protein β1 (HSPB1) to maintain mitochondrial morphology and control mitochondrial autophagy. Diabetic myocardial I/R rats showed significant improvements in cardiac function after drug treatment, while HMGB1 expression was significantly decreased, suggesting that HMGB1 could promote myocardial I/R injury in diabetic mice, and the mechanism is closely associated with its mediation of mitochondrial autophagy (49). Liu showed that by downregulating HMGB1 levels and inhibiting NF-κBp65 phosphorylation, the drug was able to improve the diabetes-induced reductions in diastolic and systolic cardiac systolic function and conduction abnormalities and alleviate cardiac insufficiency in diabetic heart disease (50). It has also been demonstrated that HMGB1 is associated with DKD cardiac function, and HMGB1 is negatively correlated with LVEF, which is an indicator of ventricular systolic function, and positively correlated with LVEDD and LVESD, which are indicators of early cardiac diastolic function. Using regression analysis, HMGB1 was shown to have the greatest effect on cardiac function in patients with DKD, and the mechanism may involve HMGB1 binding to its ligand, promoting the release of inflammatory factors, and participating in the progression of cardiac failure in patients with DKD (1).

Chronic inflammation is widely involved in DR and its complications, and HMGB1 is a key mediator of aseptic inflammation in the retina (51). HMGB1 is synthesized and secreted by immune cells, and in response to hyperglycemia or other stressors, HMGB1 binds TLR4 and RAGE. HMGB1 binds to TLR4 and RAGE, transferring the signal to the cytoplasm and inducing inflammation (52). In addition, studies have shown a significant association between disease severity and elevated HMGB1 levels (53). Moreover, researchers have constructed diabetic rat models and found that diabetes increases HMGB1 levels, which are reduced when the HMGB1 inhibitor glycyrrhizin is administered, and apoptosis, inflammatory responses and retinopathy are reduced (54). In addition, HMGB1 plays multiple roles in the inflammatory response in DN; some studies have reported that HMGB1 plays an important role in the development of DN, lupus nephritis, ANCA-associated vasculitis kidney damage, acute kidney injury and interstitial nephritis (55, 56). Zhang et al. demonstrated that the HMGB1 inhibitor glycopyrrolate ameliorated renal injury in diabetic rats, and the mechanism may be associated with the regulation of RAGE/TLR4-related ERK and p38MAPK/NF-κB-mediated inflammatory responses (57).

### 3.2 HMGB1 regulates autophagy

HMGB1 plays a pivotal role in autophagy induction and can activate autophagy in multiple ways depending on its location. Studies have suggested that HMGB1 can regulate the onset of cellular autophagy and influence apoptosis (58). HMGB1 can induce autophagy and apoptosis, promoting cell death. In fact, one study showed that HMGB1 overexpression increased autophagy and apoptosis in cardiac myocytes, ultimately resulting in cardiac injury (59). Additionally, Hu et al. demonstrated that HMGB1 overexpression promoted the development and progression of liver injury by increasing autophagy and apoptosis (60). Moreover, HMGB1 has been shown to activate the MAPK signaling pathway, leading to the phosphorylation of Bcl-2 and its segregation from Beclin1, thereby regulating the onset of autophagy. In contrast, Jin et al. found that HMGB1 enhanced autophagy by inhibiting the Akt/mTOR signaling pathway, which ultimately increased apoptosis and worsened diabetes (61).

HMGB1 has been shown to inhibit autophagy and apoptosis. Zhang et al. altered HMGB1 expression and observed reduced expression of Beclin1, LC3-II, Bax, and cleaved caspase-3 and a significant increase in the expression of LC3-I and Bcl-2. These results suggest that inhibiting HMGB1 can inhibit neuronal cell autophagy and apoptosis in the brain tissue around hematomas in rats with cerebral hemorrhage, plays dual regulatory roles in tissue injury and repair (22).

The regulation of autophagy and apoptosis by HMGB1 is important for cell survival. Jeon et al. (62) showed that HMGB1 promoted scar formation by enhancing autophagy and inhibiting apoptosis, as demonstrated by the increased expression of signaling molecules such as ERK1/2, AKT, and NF-κB in fibroblasts. Conversely, decreased autophagy and increased apoptosis were observed after the inhibition of HMGB1 expression by glycyrrhizin. This finding suggests that signaling molecules such as ERK1/2, Akt, and NF-κB may also be involved in the regulation of autophagy and apoptosis by HMGB1. The interaction between autophagy and apoptosis is regulated by many molecules, and the regulatory effect of HMGB1 on autophagy and apoptosis varies may depend on its redox status. However, the exact mechanism of action is still unclear, and further experimental studies are needed in the future (63).

### 3.3 Autophagy regulates HMGB1

**Figure 3:**
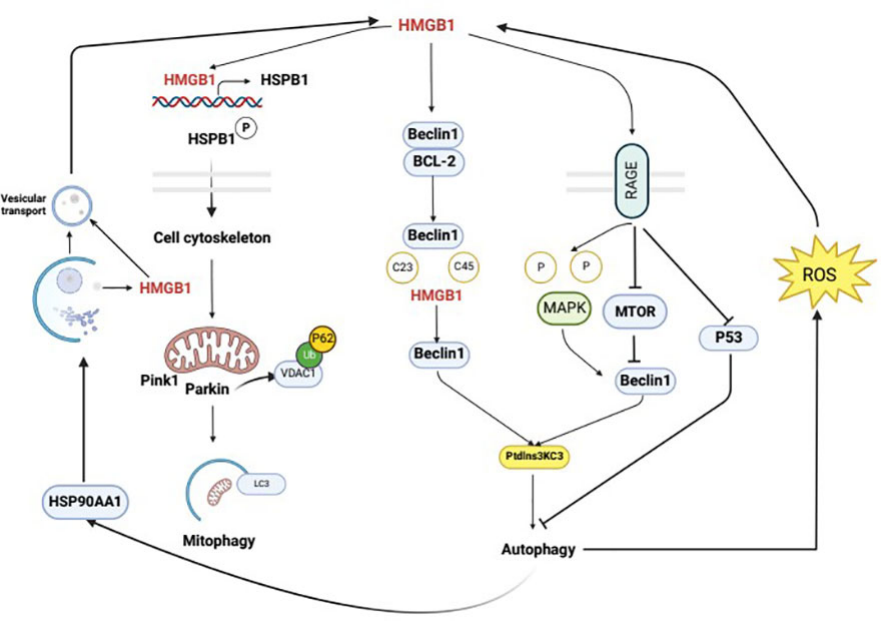
Mutual regulation mechanism of autophagy and HMGB1. Intracellular HMGB1 regulates cellular autophagy by binding with Beclin1. In the cytoplasm, HMGB1 mainly binds to Beclin1 to promote the dissociation of Beclin1 and anti-apoptotic factor Bcl-2, leading to the activation of autophagy through the interaction between Beclin1 and PI3K ClassIII/Vsp34. The specific mechanism of this process is that the semi-cysteine residues at positions 23 and 45 of the A-box of HMGB1 can form disulfide bridges with Beclin1, which results in the dissociation of Beclin1 from Bcl-2. HMGB1 regulates cellular autophagy by binding with cell membrane receptor RAGE, which can be actively secreted or passively released into the extracellular space during cell death. In the extracellular space, HMGB1 regulates cellular autophagy mainly by binding with RAGE. In the nucleus, HMGB1 participates in regulating the expression of heat shock protein B1 (HSPB1). The activation of phosphorylated HSPB1 at positions 15 and 86 by HMGB1 has an important effect on the polymerization and reorganization of the cytoskeleton, which is crucial for intracellular substance transport. HMGB1 is mainly located in the cell nucleus, and the HSP90AA1-dependent transport is involved in the nuclear-cytoplasmic translocation of HMGB1. When HMGB1 translocates to the cytoplasm, its secretion requires autophagy and vesicular transport, which can be enhanced by HSP90AA1. Upon external stimulation, autophagy can promote the translocation of HMGB1 from the nucleus to the cytoplasm through a ROS-dependent pathway, thereby replacing Bcl-2 and binding with Beclin1, which activates autophagy and further maintains it.

The interaction between HMGB1 and autophagy is bidirectional. HMGB1 may function as an autophagy activator, and autophagy, in turn, can regulate the generation, secretion, and degradation of HMGB1. Studies have shown that autophagy controls the characteristics of dying cells by regulating the selective release of HMGB1, which is thought to be associated with cell death. It is also thought that HMGB1 release may occur when apoptotic cells have high levels of autophagy (64). Additionally, Shang conducted preliminary experiments and found that an increase in autophagy may promote HMGB1 secretion and facilitate pyroptosis (65). Furthermore, when cells are stimulated by external factors, autophagy can promote the translocation of HMGB1 from the nucleus to the cytoplasm through a ROS-dependent pathway, thereby replacing Bcl-2 and binding with Beclin1 to activate the autophagic response and further maintain autophagy. In response to the autophagy inducer rapamycin, the secretion of HMGB1 into the extracellular space is increased, and conversely, the secretion of HMGB1 into the extracellular space is inhibited by the autophagy inhibitor 3-MA (47). Wang’s study first demonstrated that the secretion of HMGB1 is regulated by an autophagy-based secretion mechanism mediated by heat shock protein 90 alpha family class A member 1 (HSP90AA1) and Golgi reassembly stacking protein 2 (GORASP2). HMGB1 is mainly located in the nucleus, and HSP90AA1-dependent transport is involved in the nuclear-cytoplasmic translocation of HMGB1. When HMGB1 translocates to the cytoplasm, its secretion requires both autophagy and vesicular transport, and HSP90AA1 enhances autophagy and vesicular transport secretion mechanisms, confirming that HMGB1 is contained within autophagosomes (11). In addition, there is a close relationship between HMGB1 and NF-κB, and HMGB1 can affect the activation of NF-κB, thereby influencing the development of inflammation (66). Autophagy is also closely associated with NF-κB; these factors share common upstream signaling regulation and autophagy can inhibit the activation of NF-κB and limit the occurrence of inflammation (67). At the molecular level, autophagy and NF-κB mutually influence each other through positive and negative feedback to maintain cellular homeostasis (68). Therefore, autophagy may affect the HMGB1 pathway through the NF-κB pathway. These results suggest that there is an interaction between HMGB1 and autophagy (**Figure 3**).

## 4 Relationship between HMGB1, autophagy and diabetes

### 4.1 Diabetes and insulin resistance

**Figure 4:**
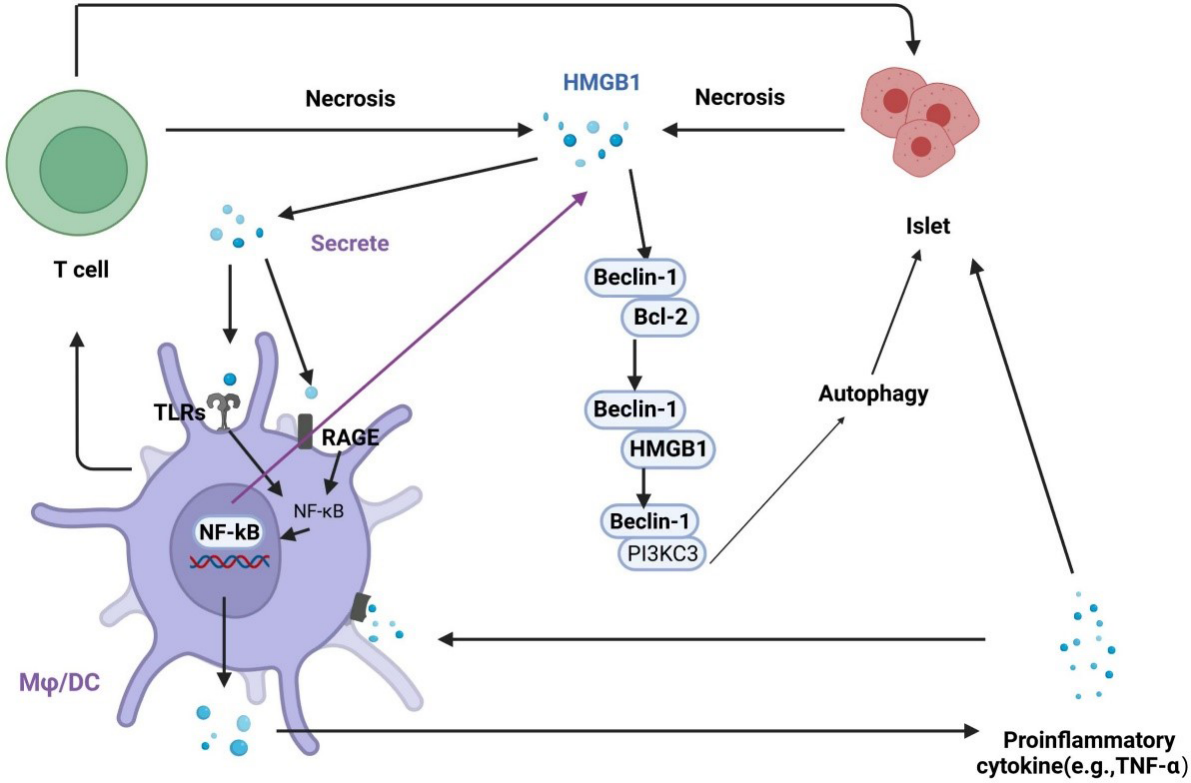
Regulation of the interplay between autophagy and HMGB1 in islet injury. HMGB1 promotes early inflammation in diabetes and leads to necrosis in islet cells. Necrotic cells release HMGB1, which activates TLRs and RAGE on macrophages and dendritic cells. The activation of TLRs and RAGE leads to the translocation of NF-κB into the nucleus to promotes the expression of inflammatory genes, which contributes to the secretion of proinflammatory cytokines, including HMGB1. In addition, activated macrophages and dendritic cells actively secrete HMGB1, exacerbating islet cell necrosis. HMGB1 can competitively interact with Beclin-1 to disrupt the Beclin-1/Bcl-2 interaction, which promotes autophagy.

HMGB1 and autophagy are closely associated with diabetes and its complications. HMGB1 is an important molecule in autophagy activation, and autophagy can regulate the production, secretion, and degradation of HMGB1 (63). Dysfunctional mitochondrial autophagy involving HMGB1 is associated with metabolic diseases. The effects of autophagy can be protective or harmful, depending on the cell type and the environment. HMGB1 interacts with Beclin1 and Atg5, key components in autophagy initiation and nucleation (69). Studies have shown that the release of HMGB1 may cause necrosis in islet β cells induced by IL-1β and may lead to diabetes onset (43). Hyperglycemia-mediated oxidative stress promotes the expression of HMGB1 and RAGE, which activate autophagy (70). Autophagy is associated with diabetes-induced organ damage, and the increased expression of HMGB1 leads to excessive autophagy, resulting in a systemic inflammatory response and organ disorders (71). Elevated serum levels of HMGB1 increase the interaction of HMGB1 with TLR4 and RAGE, which enhance the activity of apoptotic and autophagic signaling pathways (72). Chung et al. found that inhibiting HMGB1 in pancreatic islet β cells promoted apoptosis, inhibited autophagy, and led to cell dysfunction (73). These results suggest that there is a strong correlation between HMGB1 and autophagy in the progression of diabetes (**Figure 4**).

Research has demonstrated that HMGB1-mediated autophagy exerts distinct effects across diverse pathologies. Specifically, Chen observed that inhibiting autophagic flux mitigated HMGB1 levels, culminating in the alleviation of myocardial ischemia-reperfusion damage in diabetic mouse models (74). Conversely, Chung used englitazone to suppress HMGB1 expression, thereby impairing autophagy and disrupting cellular function, ultimately inducing diabetes (73). Additionally, Wu revealed that increased glucose levels stimulated autophagy, and inhibiting HMGB1 expression attenuated autophagy levels (75).

HMGB1 and autophagy are closely linked to diabetes and its complications. HMGB1 can act as an autophagy activator, and autophagy can regulate the generation, secretion, and degradation of HMGB1. Mitochondrial autophagy dysfunction involving HMGB1 is associated with metabolic diseases such as diabetes and fatty liver. Autophagy can be protective or harmful, depending on the cell type and the environment. HMGB1 interacts with key components of autophagy initiation and nucleation, including Beclin1 and Atg5 (76).

Studies have shown that IL-1β-induced pancreatic β cell death and HMGB1 release may lead to the onset of diabetes (43). Hyperglycemia-mediated oxidative stress promotes the expression of HMGB1 and RAGE, thereby activating autophagy (70). Autophagy is associated with organ damage in diabetes, and increased HMGB1 expression leads to excessive autophagy, which in turn causes systemic inflammation and organ dysfunction (71). In the serum of diabetic rats, the level of HMGB1 is elevated, which is accompanied by an increase in the interactions of HMGB1 with TLR4 and RAGE, enhancing the activity of apoptosis and autophagy signaling pathways (72). Chung et al. found that inhibiting the expression of HMGB1 in pancreatic β cells promoted apoptosis, inhibited autophagy, reduced cell survival under stress conditions, caused cell dysfunction, and led to diabetes (73).

Insulin resistance is the main cause of the occurrence and development of diabetes. The occurrence and development of insulin resistance are closely associated with two signaling pathways: JNK and NF-κB. Autophagy is closely associated with the NF-κB pathway, and they share many common regulatory signaling factors. Autophagy inhibits the activation of NF-κB and limits the occurrence of inflammation (67). Autophagy and NF-κB affect each other through positive and negative feedback to maintain intracellular stability (68). Therefore, autophagy may interact with NF-κB through HMGB1.

The serum level of HMGB1 can be used as a biomarker of glucose toxicity, atherosclerosis, and hypoβ-cell function in diabetic patients. Serum HMGB1 concentrations are positively correlated with glucose metabolism indices such as HbA1c and FPG (77). HMGB1 increased the expression of ATG7 and the LC3B II/I ratio, decreased the expression of p62, and intensified autophagy (78). In addition, it has been reported that serum HMGB1 levels are positively correlated with FINS and HOMA-IR and negatively correlated with HOMA-β, suggesting that HMGB1 may impair β pancreatic cell function and increase insulin resistance (77). HMGB1 accelerates insulin resistance by increasing the expression of RAGE, activating the TLR4/JNK/NF-κB pathway and reducing activation of the IRS-1 signaling pathway (79, 80).

### 4.2 Diabetic complication

#### 4.2.1 Diabetic cardiomyopathy

DCM is one of the common complications of diabetes and is the leading cause of death in diabetic patients. HMGB1 is involved in the occurrence and development of DCM. Current research suggests that diabetic myocardial ischemia/reperfusion injury and increases in oxidative stress and ROS are closely associated with mitochondrial dysfunction. Some scholars consider diabetic heart disease to be a mitochondrial disorder (48). HMGB1 is a key regulator of mitochondrial autophagy, and oxidative-reductive reactions can induce HMGB1 translocation, leading to sustained activation of proinflammatory pathways and enhanced myocardial injury through binding with RAGE. HMGB1 also regulates HSPB1 to maintain mitochondrial morphology and control mitochondrial autophagy, playing an important role in myocardial remodeling after diabetic I/R (81). Excessive autophagy during ischemia/reperfusion in DCM can exacerbate myocardial injury (82). Studies have shown that reducing HMGB1 expression in DCM rats can reduce infarct volume, improve hemodynamics, and alleviate inflammation (83). Inhibiting HMGB1 can reduce myocardial ischemia/reperfusion injury by suppressing autophagy (72). Wu et al. found that HMGB1 promoted myocardial I/R injury in diabetic mice, and the mechanism as closely associated with the mediation of mitochondrial autophagy. Neutralizing antibodies against HMGB1 can effectively inhibit the release of HMGB1 from the heart, reduce the LC3-II/I ratio, suppress mitochondrial autophagy, and alleviate myocardial injury in diabetic mice (84). HMGB1 also regulates HSPB1 to maintain mitochondrial morphology and control mitochondrial autophagy, which plays an important role in myocardial remodeling after diabetes (81). Therefore, anti-HMGB1 therapy is a promising new approach that can reduce HMGB1-induced inflammatory damage, inhibit autophagy, and improve patient prognosis.

#### 4.2.2 Diabetic retinopathy

With the increasing incidence of diabetes, the rate of blindness caused by DR is also increasing yearly. Recent studies have shown that autophagy is associated with the mechanism of pathological neovascularization and neurodegeneration mediated by HMGB1, which is involved in the occurrence and development of DR (85, 86). The role of autophagy in DR is fairly complex. In the early stage of DR, autophagy can promote cell survival, while excessive autophagy will lead to necrosis and exacerbate DR. Intermittent hyperglycemic oxidative stress can regulate autophagy in retinal pigment epithelial cells and promote cell survival by increasing HMGB1 (87). HMGB1 can enter the lysosome through the autophagy-lysosome pathway, and lysosomal enzyme B, an important member of the autophagy pathway, is released from the lysosome into the cytoplasm, inducing inflammation and apoptosis (88). Feng et al. used retinal pigment epithelial (RPE) cells to investigate the relationship between HG and the autophagy-lysosome pathway and found that HMGB1 participated in lysosomal membrane penetration (LMP) and autophagy inhibition. The decrease in HMGB1 expression restored autophagic degradation, reduced inflammatory cytokine and VEGF expression, and protected RPE cells in the early stage of DR (89).

#### 4.2.3 Diabetic nephropathy

Podocyte injury characterized by hypertrophy, apoptosis, and epithelial-mesenchymal transformation (EMT) is the main cause of DN. Previous studies have reported that autophagy dysfunction is one of the main risk factors for podocyte injury, and HMGB1 is involved in the development of DN through autophagy (90). Inhibiting HMGB1 reduced apoptosis and injury in podocytes and delayed the deterioration of glomerular function caused by diabetes by activating the Akt/mTOR signaling pathway and inhibiting autophagy (61).

#### 4.2.4 Diabetic neuropathy

The longterm hyperglycemic environment of diabetes causes metabolic disorders and microcirculation disorders, resulting in neuronal ischemia and hypoxia and a series of nervous system diseases. As a proinflammatory mediator, HMGB1 is involved in diabetic neuropathy by binding with RAGE and TLR4 and regulating autophagy. Elevated levels of HMGB1 stimulate glutamate release and mediate neurotoxicity (91). Neuronal cells release HMGB1 during seizures, and TLR4 expression also increases (92). Guo et al. subjected KKAy mice to intermittent hypoxia to establish a diabetic neuropathy model and found that the protein expression levels of HMGB1 and TLR4 were increased and neuronal apoptosis was exacerbated. Furthermore, HMGB1 siRNA can significantly reduce the protein expression of HMGB1 and TLR4 to regulate autophagy and reduce neuronal apoptosis (93).

#### 4.2.5 Diabetic foot disease

Diabetic foot disease is mainly caused by changes in blood vessels and nerves and is a relatively common complication of diabetes. HMGB1 can amplify the inflammatory response and lead to the accumulation of macrophages, thus promoting atheromatous formation. A clinical study showed that diabetic foot patients have higher HMGB1 expression, and their arteries become narrow. HMGB1 also increased inflammation and oxidative stress and increased the expression of autophagy proteins (94).

## 5 Regulating HMGB1 to treat diabetes

In recent years, studies on the treatment of diabetes and its complications by modulating HMGB1 with traditional Chinese medicine (TCM) are increasing and becoming a research hotspot. The mechanism mainly involves the inflammatory response, oxidative stress, and autophagy, providing a theoretical and experimental basis for the prevention and treatment of diabetes and its complications (**Table 1**).

**Table 1:** TCMs that diabetes through the regulation of HMGB1.

Names	Model	Dose	Disease	Pathological mechanisms	Ref
Glycyrrhizin	Wistar rats+STZ (30 mg/kg)	200 mg/kg/d (by gavage)	DCM	HMGB1/NF-κB	95
	Mouse corneal epithelium (MCEC)+HG (25 mM), C57BL6/J +STZ (60 mg/kg)	1 mM/24 h, 150 mg/kg/d (by gavage)	Diabetic cornea	HMGB1, inflammation, oxidative stress	96
	C57BL6/J +STZ (60 mg/kg)	150 mg/kg/d (by gavage)	DR	HMGB1, inflammation	97
	C57BL6/J +STZ (60 mg/kg)	150 mg/kg/d (by gavage)	Diabetes-induced neuronal and vascular damage	HMGB1/SIRT1	98
	Zucker diabetic fatty (ZDF) rats	50 mg/kg/d (i.p.)	DN	HMGB1/TLR4/NF-κB	99
	KK/TaJcl mice+HFD	10 mg/kg/d (i.p.)	Diabetic periodontitis	HMGB1/RAGE	100
Astilbin	SD rats+STZ (50 mg/kg)+ myocardial I/R injury, H9C2 cells (hypoxia model)	1.5, 5, 15, 50 μM/24 h, 50 mg/kg/d (i.v.)	DCM	HMGB1/NF-κB	83
Albiflorin	SD rats+STZ+HFD	100, 200 mg/kg/d (by gavage)	Diabetic cognitive impairment	Nrf-2/HO-1/HMGB1/NF-κB	101
Dihydromyricetin	SD rats+STZ (30 mg/kg)+ HFD	250 mg/kg/d (by gavage)	DCM	HMGB1/NF-κB	50
	HUVECs+HG (30 mmol/L)	0.1, 1, 10 μmol/L/24 h	Endothelial dysfunction in DM	HMGB1/NF-κB, inflammation	102
Paeonol	SD rats+STZ (60 mg/kg), corpus cavernosum smooth muscle cells (CCSMCs)+HG (30 mM)	100 mg/kg/d (by gavage), 100 μM	Diabetic erectile dysfunction	HMGB1/RAGE/NF-κB	103
Formononetin	C57BL/6J+STZ (180 mg/kg), SH-SY5Y+HG	25, 50 mg/kg/d (by gavage), 2.5, 5, 10 μM/22 h	Diabetic cognitive impairment	HMGB1/TLR4/NF-κB, NLRP3 inflammasome	104
Bupleurum polysaccharides	C57BL/6J+STZ (100 mg/kg)	30, 60 mg/kg/d (by gavage)	DN	HMGB1/TLR4	105
Matrine	Mouse podocytes+HG (30 mM)	0.5, 1.0 mg/mL/48 h (by gavage)	DN	HMGB1/TLR4/NF-κB	106
Salidroside	db/db mice	40, 80 mg/kg (by gavage)	Insulin resistance and liver injury	HMGB1/RAGE/NF-κB, HMGB1/TLR4/NLRP3	107
Rhodiola crenulata	HUVECs+HG (33 mM)	1.5, 3, 15, 30 μg/mL (by gavage)	Endothelial dysfunction in DM	HMGB1/TLR4	108
Polygonum cuspidatum	SD rats+STZ (60 mg/kg)	100, 350 mg/kg/d (by gavage)	DR	HMGB1/RAGE/NF-κB	109
Yiqi Tongluo Formula	SD rats+STZ (55 mg/kg)	1.84, 7.36 g/kg/d (by gavage)	Diabetic peripheral neuropathy	HMGB1	110
Jinkui Shenqi Decoction	SD rats+ HFD+STZ (40 mg/kg)	0.9, 3.6, 1.8 g/kg/d (by gavage)	DN	HMGB1, Beclin 1	90
Puerariae Lobatae Radix and Anemarrhenae Rhizoma	SD rats+ HFD+STZ (35 mg/kg)	4.62, 9.24 g/kg/d (by gavage)	Diabetic cognitive impairment	HMGB1/RAGE/NF-κB	111
Tongluo Xiaoke Formula	Wistar rats+STZ (55 mg/kg)	1.84, 7.36 g/kg/d (by gavage)	DCM	HMGB1/TLR4	112

### 5.1 The active ingredients or extracts of TCM

Glycyrrhizin (GR) is a natural compound extracted from *Glycyrrhiza glavra* that has been reported to exert anti-inflammatory, immunomodulatory, antidiabetic, antitumor, anti-infective, antioxidant, and hematopoietic effects (113). GR can alleviate DCM by inhibiting HMGB1 expression in myocardial tissue, which inhibits activation of the NF-κB inflammatory signaling pathway and the release of inflammatory factors and attenuates cardiomyocyte apoptosis (95). GR has also been shown to ameliorate DR. Mallika et al. demonstrated that GR increased cell viability and decreased the inflammatory and oxidative stress molecules HMGB1, IL-1 β, TLR2, TLR4, NLRP3, COX2, SOD2, HO-1, gpx2, and GR1 in an HG environment (96). Liu et al. showed that GR improves vascular permeability by inhibiting inflammation, protecting neurons, and reducing vascular damage (97). GR protects the retina by increasing SIRT1-mediated inhibition of HMGB1 and decreasing TNF-α and IL-1β levels in a mouse model of DR (98). In addition, GR could alleviate glomerular injury in DN rats by reducing HMGB1 expression and inhibiting the NF-κB and TLR4 pathways (99). Keiichi et al. found that licorice sweetener inhibited periodontal and systemic inflammation in diabetic mice and reduced blood glucose levels *via* the HMGB1-RAGE axis (100).

Astilbin, a flavonoid compound isolated from the rhizome of *Smilax china* L, has been reported to have an anti-inflammatory effect (114). Diao et al. established a rat model of ischemia-reperfusion injury in DCM and administered astilbin. The results showed that 50 mg/kg astilbin reduced infarct volume, improved hemodynamics, reduced myocardial injury, and decreased serum proinflammatory factor levels. The mechanism mainly involved inhibiting HMGB1 expression and the NF-κB signaling pathway to block the myocardial inflammatory cascade response (83).

Albiflorin is a natural compound extracted from the roots of Radix Paeoniae Alba, has significant antidepressant effects and is a potential treatment for diabetes (115). Ma et al. found that albiflorin treatment improved behavioral and hippocampal pathological changes in cognitively impaired diabetic rats. The main mechanism involved improving oxidative stress and inflammation by modulating the Nrf-2/HO-1/HMGB1/NF-κB signaling pathway (101).

Dihydromyricetin is the most abundant natural flavonoid isolated from *Ampelopsis grossedentata*. It has strong anti-inflammatory and protective effects on diabetic heart dysfunction (116). Dihydromyricetin has been shown to ameliorate reductions in diastolic and systolic function and conduction abnormalities induced by diabetes by downregulating HMGB1 and inhibiting the phosphorylation of NF-κB p65 (50). The same result was found *in vitro*: dihydromyricetin reduced hyperglycemic-induced apoptosis in vascular endothelial cells by inhibiting HMGB1/NF-κB (102).

Paeonol is a natural compound extracted from Moutan Cortex. Paeonol can alleviate DM-induced erectile dysfunction in rats, improve fibrosis and pathological changes, and reduce apoptosis and inflammation in corpus cavernosum smooth muscle cells (CCSMCs) under HG conditions by downregulating the HMGB1/RAGE/NF-κB pathway (103).

Formononetin improved cognitive impairment in STZ-induced diabetic rats, enhanced learning and memory abilities, and downregulated the levels of HMGB1, TLR4, MyD88, p-IκB, p-NF-κB, NLRP3, SOD, MDA, TNF-α, IL-1β, and IL-6. The mechanism may involve inhibiting HMGB1/TLR4/NF-κB signaling and the NLRP3 inflammasome (104).

Bupleurum polysaccharides alleviated urinary albumin and creatinine levels and alleviated renal histopathological damage in diabetic mice. The mechanism involved inhibiting the production of type IV collagen, fibronectin, and α-smooth muscle actin, as well as reducing the levels of inflammatory factors, and inflammation and fibrosis in the kidney by blocking the HMGB1/TLR4 pathway (105).

Matrine is a component of *Sophora flavescens* Root that has favorable medicinal effects. Matrine can alleviate HG-induced podocyte apoptosis by inhibiting the inflammatory cytokines IL-1β, IL-6 and TNF-α by inhibiting the HMGB1-associated TLR4/NF-κB pathway (106).

Salidroside is the primary active ingredient of Rhodiola and can alleviate insulin resistance, hyperglycemia, and liver inflammation in db/db mice by inhibiting the HMGB1/RAGE/NF-κB and HMGB1/TLR4/NLRP3 signaling pathways (107). Rhodiola crenulata protects against HG-induced oxidative stress injury in cultured human umbilical vein endothelial cells (HUVECs). Rhodiola crenulata reduced the levels of ROS and HMGB1, inhibited the TLR4/MyD88 signaling pathway, reduced the expression of intracellular adhesion molecule-1 (ICAM-1) and vascular adhesion molecule-1 (VCAM-1), and alleviated apoptosis in endothelial cells (108).

Polygonum cuspidatum could reduce the expression of HMGB1 and RAGE in the retinal tissues of diabetic retinal rats, inhibit the NF-κB pathway, improve retinal vascular permeability and tight junction protein expression, and inhibit the development of DR (109).

### 5.2 TCM formulas

Taohong Siwu Decoction is composed of Persicae Semen, Angelicae Sinensis Radix, Carthami Flos, Chuanxiong Rhizoma, Rehmanniae Radix Praeparata and Paeoniae Radix Alba, which can reduce the level of HMGB1, alleviate insulin resistance and reduce inflammation in T2DM patients (87). Yiqi Tongluo Formula is an effective herbal prescription composed of Astragalus membranaceus (Fisch.) Bunge, Radix Puerariae, Cinnamomum cassia Presl, Angelicae Sinensis Radix, Rehmannia glutinosa (Gaertn.), and Pheretima, which can treat diabetic neuropathy by reducing inflammation and downregulating the level of HMGB1 (110). Jinkui Shenqi Decoction is composed of Rehmannia glutinosa (Gaetn.), Dioscorea opposita Thunb., Cornus officinalis Sieb., Cinnamomum cassia Presl, Aconitum carmichaeli Debx., Alisma orientale and Cortex Moutan. It exerts a therapeutic effect on DN rats by inhibiting the protein expression of HMGB1, TLR4, and p62 and promoting Beclin 1-mediated autophagy (90). The water extracts of Pueraria has a significant therapeutic effect on a rat model of diabetic cognitive dysfunction by lowering the level of blood sugar and glycosylated hemoglobin and ameliorating cognitive dysfunction and memory impairment. The mechanism involves downregulating the HMGB1/RAGE/NF-κB pathway (111). Tongluo Xiaoke Formula is composed of Astragalus membranaceus (Fisch.), Puerariae Lobatae Radix, Rehmannia glutinosa (Gaertn.), Cinnamomum cassia Presl, Angelicae Sinensis Radix, Schisandra chinensis, and Lumbricus. Low and high doses of Tongluo Xiaoke Formula can reduce cytokines and inflammatory mediators by downregulating the expression of HMGB1 and TLR4 (112).

### 5.3 The impact of chemical medicines on the treatment of diabetes by regulating HMGB1

T2DM is a prevalent metabolic disease that is often accompanied by abnormal lipid metabolism and contributes to cerebrovascular diseases such as cerebral infarction (117). Recent research has shown that the combination of sodium ozagrel and atorvastatin can effectively reduce the inflammatory response, regulate HMGB1 levels, and regulate blood glucose and lipid levels in patients with T2DM and lacunar infarction (118). Another complication that commonly arises in diabetic patients is DN. Englazine, a sodium-glucose cotransporter-2 (SGLT-2) inhibitor, has anti-inflammatory and antioxidant effects and can lower the level of HMGB1 and alleviate kidney inflammation in diabetic rats (119). Additionally, dagliazine has been shown to exert therapeutic effects on renal inflammation, lipid deposition, oxidative stress, and fibrosis by inhibiting HMGB1 expression and stimulating autophagy in diabetic mice, thereby providing a new therapeutic target for DN (120). Furthermore, the combination of sitagliptin tablets and Huangkui capsules has been shown to effectively reduce blood sugar, improve renal function, and lower the level of inflammatory factors, such as HMGB1, in early DN patients (121). Collectively, these findings offer promising strategies for managing the complications associated with T2DM.

## 6 The treatment of diabetes through the regulation of autophagy

Based on the mechanism of autophagy, TCM has shown significant therapeutic efficacy in treating diabetes and its complications, particularly DCM and nephropathy. Individual active compounds and TCM formulas can promote or inhibit autophagy in different diseases. The main targets are Beclin1, LC3-II, mTOR, TGF-β1, α-SMA, and PI3K/Akt/mTOR.

### 6.1 Promoting autophagy with Chinese herbal monomers and formulas

Kaempferol is a flavonoid found in TCMs such as lotus leaf, ginkgo biloba, Puhuang, and Danhong. In PA-induced RIN-5F pancreatic β cells, kaempferol increased the number of autophagosomes and upregulated the gene expression for Beclin-1, ULK1, ATG5, ATG7, and LC3b. The expression level of LC3-II protein was increased, and the ratio of LC3-II/LC3-I was increased, while the expression level of p62 protein decreased, indicating that kaempferol could induce autophagy in PA-induced pancreatic β cells (122). Additionally, berberine, an alkaloid extract from Coptis chinensis, can activate AMPK and promote autophagy in pancreatic β cells (123).

Autophagy is an important pathological mechanism in DCM and can be regulated by various TCM compounds, single herbs, and active components. The progression of DCM is closely associated with abnormal autophagy in cardiomyocytes, and treatment focuses on restoring normal autophagy in these cells. TCM can promote autophagy in DCM through the AMPK/mTOR signaling pathway. Compounds such as crocin (124), Astragalus polysaccharide (125), curcumin (126), and tea polyphenols (127) can increase AMPK expression by promoting AMPK phosphorylation in cardiomyocytes. This leads to the inhibition of mTOR and its downstream effectors p70S6K1 and 4EBP1, which in turn increases autophagy in myocardial cells. Additionally, asiaticoside (128) can enhance autophagy in cardiomyocytes by inhibiting the NOTCH1/HES1 signaling pathway, thereby improving myocardial injury induced by DCM. Zhi Gancao decoction, which is composed of 12 g of Glycyrrhiza uralensis Fisch, 9 g of Zingiber officinale Rosc. and Cinnamomum cassia Presl, 6 g of Ginseng Radix et Rhizoma, 50 g of Rehmannia glutinosa Li-bosch., 6 g of Colla Corii Asini, 10 g of Ophiopogon japonicus, 10 g of Cannabis sativa L., and 10 g of Ziziphus zizyphus, can downregulate the protein expression of P62 in high-fat diet- and STZ-induced DCM model rats. Moreover, Zhi Gancao decoction can upregulate the expression of LC3-II/LC3-I and Beclin-1, enhancing autophagy in cardiomyocytes (129).

Autophagy plays a crucial role in the pathogenesis of DN, and numerous TCMs have been shown to regulate autophagy *via* the AMPK/mTOR signaling pathway to treat DN. Saponins, which are steroidal compounds, are the active constituents of many TCMs. Zhang (130) investigated the effects and mechanism of nosaponin on DN in SD rats and found that it significantly improved kidney pathology by reducing glomerular volume, the mesangial matrix area, fibronectin levels, and p-mTOR/mTOR expression levels. In addition, nosaponin significantly increased Beclin-1, LC3-II/LC3-I, and p-ULK1/ULK1 levels, indicating that nosaponin could promote autophagy and protect the kidneys by activating the AMPK/mTOR signaling pathway. The alkaloid berberine is another TCM that regulates autophagy in DN. Yan et al. (131) showed that berberine upregulated autophagy, reduced damage to the McP-5 murine immortalized podocyte line in a HG environment, and enhanced the protective effect on DN podocytes. Animal studies have also demonstrated that berberine upregulates autophagy-related protein expression levels and regulates autophagy levels, thereby protecting against kidney injury (132). Modified Jisheng Shenqi Decoction, which consists of cinnamon Cinnamomum cassia Presl (6 g), Aconiti Lateralis Radix Praeparata (8 g), Rehmannia glutinosa (15 g), Dioscorea opposite (25 g), Cornus officinalis (15 g), Poria cocos (20 g), Alisma orientale (8 g), Cortex Moutan (8 g), Achyranthes bidentata (15 g), Semen Plantaginis (10 g), Astragali Radix (25 g), Rheum officinale baill (8 g), Amygdalus persica L (10 g), and Manis pentadactyla (25 g), could enhance autophagy in DN rats. Jiang found that modified Jisheng Shenqi Decoction significantly increased the LC3II/I ratio, decreased P62 levels, and enhanced autophagy, thereby improving the body’s ability to resist stress and slowing DN development (133).

### 6.2 Inhibiting autophagy with TCM monomers and formulations

Some TCMs have been shown to negatively regulate autophagy in pancreatic β cells. For instance, ginseng extract has been shown to reduce the formation of autophagosomes in tacrolimus (Tac)-induced mouse islet β cells and INS-1 cells, decrease the LC3B-II/LC3B-I ratio, inhibit the degradation of autophagic lysosomes, and further improve the function of islet β cells by regulating autophagy to inhibit TAC-induced oxidative stress (134). Excessive autophagy in DCM can accelerate the degradation of damaged cells and metabolites, and it can damage normal cardiomyocytes, leading to the apoptosis in many cardiomyocytes, which exacerbates myocardial hypertrophy and fibrosis. Therefore, inhibiting autophagy in cardiomyocytes may reduce the damage caused by autophagy. One way to do this is to promote the PI3K/AKT signaling pathway. For example, astragaloside (135) and irisin (136) inhibit autophagy in cardiomyocytes by activating mTOR, which is a negative regulator of autophagy in the PI3K/AKT pathway. Additionally, icarin (137) can inhibit activation of the AMPK/mTOR signaling pathway on autophagy by inhibiting the AMPK/mTOR signaling pathway, which reduces the levels of p-AMPK and increases the levels of p-mTOR in the myocardium, thereby improving autophagy in the overactivated diabetic myocardium. Cordyceps sinensis is a valuable medicinal fungus of the Cordyceps genus, which is mainly composed of amino acids, fatty acids, polysaccharides, organic acids, and a variety of trace elements. Tu (138) administered Cordyceps sinensis to Wistar rats with DN and found that it could effectively regulate the expression of the AMPK/mTOR signaling pathway, reduce the protein levels of LC-3II, and effectively reduce the occurrence of renal tubular autophagy.

Yunv Decoction consists of Gypsum Fibrosum, Radix Rehmanniae Preparata, Liriope spicata, Rhizoma Anemarrhenae and Achyranthes bidentata. He et al. administered Yunv Decoction to rats with T2DM for 4 weeks and found that it decreased the protein expression of LC3 in islets, indicating that Yunv Decoction could downregulate the level of islet autophagy (139).

### 6.3 The impact of new hypoglycemic medications on autophagy

Novel classes of drugs to treat diabetes include glucagon-like peptide-1 receptor agonists (GLP-1RAs), dipeptidyl peptidase-4 inhibitors (DPP-4is), and sodium-glucose cotransporter-2 inhibitors (SGLT2is). Shao et al. (140) evaluated the hepatoprotective effects of GLP-1RA on diabetic mice and found that it inhibited activation of the NLRP3 inflammasome *via* the mitochondrial autophagy pathway. The results showed that GLP-1RA effectively reduced fasting blood glucose (FBG), total cholesterol (TC), triglyceride (TG), and malondialdehyde (MDA) levels in mice. Shui et al. (141) treated T2DM rats with the SGLT2i empagliflozin and found that it restored the number and size of mitochondria by promoting autophagy, inhibited mitochondrial shrinkage, and improved the prognosis of myocardial infarction. GU et al. used the DPP-4i sitagliptin to activate autophagy in streptozotocin-induced diabetic mice to improve cardiac function after myocardial infarction. The results showed that sitagliptin effectively increased the survival rate, improved cardiac function, and reduced infarct size, myocardial fibrosis, and the inflammatory response (142).

## 7 Summary

In this article, we have summarized the relationship between autophagy and HMGB1 in diabetes and its complications and discussed potential therapeutic strategies to alleviate diabetes by regulating autophagy and HMGB1. Autophagy and HMGB1 play important roles in diabetes and its complications. Autophagy is related to organ damage caused by diabetes. Increased HMGB1 can increase TLR4 and RAGE, enhance the activity of the autophagy signaling pathway, and lead to dysregulated autophagy, thus causing insulin resistance, systemic inflammatory response, and organ dysfunction. HMGB1 can also regulate HSPB1 to maintain mitochondrial morphology and control mitochondrial autophagy. It is thought that HMGB1 stimulation can induce autophagy, and autophagy can also affect the expression of HMGB1. Autophagy and HMGB1 are related in several ways. HMGB1 can regulate autophagy by modulating Akt/mTOR, MAPK, ERK1/2, AKT, and NF-κB signaling molecules and pathways. Autophagy regulates the production, secretion, and degradation of HMGB1 through the ROS-dependent pathway, positive and negative feedback of HSP90AA1 and NF-κB. Meanwhile, we found that ingredients and compounds in traditional Chinese medicine (TCM) can treat diabetes by regulating HMGB1 and autophagy. These include Glycyrrhizin, Astilbin, Albiflorin, Dihydromyricetin, Paeonol, Formononetin, Salidroside, Kaempferol, Berberine, Astragalus, polysaccharide, Irisin, Taohong Siwu Decoction, Yiqi Tongluo Formula, Jinkui Shenqi Decoction, Yunv Decoction, and Zhi Gancao Decoction compound, mainly related to inflammation, oxidative stress, etc. These treatments include AMPK/mTOR inhibitors, Nrf-2, HO-1, RAGE, NF-kappa B, TLR4, SIRT1 mechanisms, and signaling molecules. Additionally, some chemical medicines such as sodium ozagrel, atorvastatin, DN. Englazine, GLP-1Ras, and DPP-4is have been shown to regulate HMGB1 and autophagy and play a role in the treatment of diabetes. Although some studies have been conducted on the role of autophagy and HMGB1 in diabetes and its complications, the pathological mechanisms of autophagy dysfunction, abnormal HMGB1 expression, and induced injury remain largely unexplored. Intervening in HMGB1 or other autophagy targets and exploring their mechanisms are of great theoretical importance and have clinical applications for the prevention and treatment of diabetes and its complications. This review can provide new directions for follow-up research and open up new research avenues for current treatments.

## Author contributions

KY and FC carried out the manuscript writing. WW added a substantial amount of content and improved the contextual connections. LY and ZT provided ideas and technical guidance for the whole work. All authors contributed to the article and approved the submitted version.
